# Endoscopic ultrasound guided therapy of gastric varices: Initial experience in the Arab world (with video)

**DOI:** 10.3389/fgstr.2022.989987

**Published:** 2022-09-21

**Authors:** Ali A. Alali, Ahmad Hashim, Asma Alkandari

**Affiliations:** ^1^Haya Al-Habeeb Gastroenterology and Hepatology Center, Mubarak Al-Kabeer Hospital, Jabriya, Kuwait; ^2^Department of Medicine, Faculty of Medicine, Kuwait University, Jabriya, Kuwait; ^3^Department of Gastroenterology, Al-Jahra Hospital, Al-Jahra, Kuwait

**Keywords:** endoscopic ultrasonography, gastric variceal bleeding, coil embolization, cyanoacrylate glue, portal hypertension

## Abstract

**Background and objectives:**

Gastric varices (GV) bleeding is a catastrophic complication of portal hypertension and is associated with significant morbidity and mortality. There are limited effective therapeutic interventions for the management of bleeding GV. Recently, EUS-guided therapy has been shown to be effective and safe intervention for such patients. However, there are no data to describe the feasibility and safety of EUS-guided therapy for GV in Arab population. The aim of this study is to describe our initial experience of EUS-guided therapy for GV in Kuwait.

**Methods:**

A case-series of patients that underwent EUS-guided therapy for clinically significant GV. All patients underwent EUS-guided therapy including Cyanoacrylate (CYA) injection with or without coil embolization. Patients were followed post procedure to document GV obliteration and monitor for any adverse events.

**Results:**

In total, 15 patients were included in this study (80% male) with mean age of 58 ± 12 years. The main indication for therapy was active GV bleeding (53.3%) followed by secondary prophylaxis (33.3%). Most patients had GOV-2 (80%) with mean GV size of 24.9 ± 7.9 mm. Combined EUS coil-CYA was used in most patients (80%), mean volume of CYA injected was 1.5 ± 0.74ml and mean number coils used of 1.5 ± 1.4. The technical success rate was 100% and all patients achieved GV obliteration after a median of 1 session (range 1-2). There were no major adverse events.

**Conclusion:**

Among Arab population with portal hypertension, EUS-guided therapy is highly effective and safe option for the managements of clinically significant GV.

## Introduction

Gastric Varices (GV) are an important cause of upper gastrointestinal bleeding (UGIB). Approximately 20% of patients with portal hypertension develop GV (1). GV can lead to more severe bleeding and is associated with higher morbidity and mortality compared to esophageal variceal bleeding (2). Furthermore, the risk or rebleeding is higher with GV ranging between 34-89% (3, 4). One of the most widely used treatment for GV is endoscopic injection of cyanoacrylate (CYA) first described by Soehendra in 1986 (5). Even though this technique has been shown to be effective with high hemostasis rate and low re-bleeding risk, it has been associated with many severe adverse events including systemic embolization, fever, chest pain and even death (6, 7).

EUS-guided therapy has recently been introduced as a more effective and safer option than endoscopic therapy for GV. EUS-guided therapy includes EUS-guided CYA injection alone or in combination with EUS-guided coiling. EUS offers the advantage of directly visualizing the varices and delivering targeted therapy. In a large cohort of 152 patients with GV who underwent EUS-guided embolization and glue injection, the obliteration rate was 93% with only 3% rebleeding rate (8). Furthermore, a recent meta-analysis has concluded that EUS-guided coiling and CYA injection achieved superior GV obliteration compared to endoscopic CYA injection alone (9).

Despite all these promising results, there are scarce data describing the efficacy of EUS-guided therapy in patient with GV in Arab population. Herein we describe our experience in patients with GV undergoing EUS-guided therapy in Kuwait.

## Patients and methods

This was a case series of patients undergoing EUS-guided therapy for GV performed in Haya Al-Habeeb gastroenterology center in Mubarak Al-Kabeer hospital in Kuwait from October 2017 to October 2021. Procedural and clinical data were collected from the hospital electronic medical record. Ethical approval for the study was obtained from the Institutional Review Board (IRB).

All procedure were performed in the endoscopy unit by one endoscopist (AA). Procedures were done under either conscious sedation (midazolam and fentanyl) or general anesthesia with endotracheal intubation. Informed consent was obtained from all patients before the procedure. Patients were placed on the left lateral position during the procedure. Prophylactic antibiotics (ceftriaxone 1g IV) was given selectively to patients with active GI bleeding only. The EUS-guided procedure was performed using the linear-array therapeutic echoendoscope (3.8mm working channel; EG3870UTK; Pentax, Hamburg, Germany) attached to an ultrasound processor (Hitachi, Tokyo, Japan).

**Figure 1:**
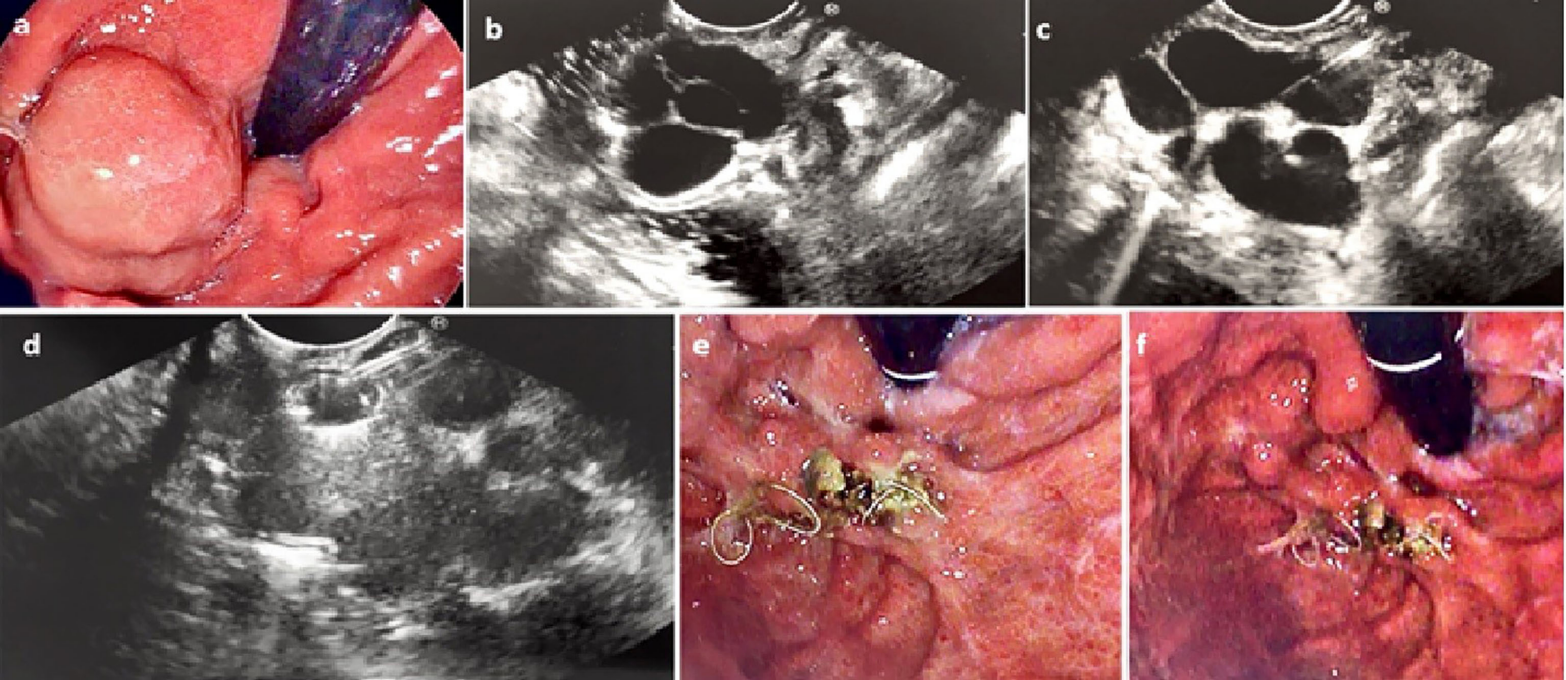
**(A)** Endoscopic assessment of a large IGV-1. **(B)** Assessment by EUS confirming large (2.6cm) intramural GV **(C)** The GV was punctured by 19G FNA needle **(D)** Coil deployed into the GV via the 19G FNA needle under direct EUS vision **(E)** and **(F)** Repeat endoscopic assessment 6 weeks later confirmed eradication of the GV with coil seen extruding from the area of the treated GV.

Initially the GVs were assessed using regular gastroscope to assess for any active bleeding or stigmata of recent bleed (red wale sign, ulceration at the GV, or platelet plug). Next, the echoendoscope was introduced and water was instilled into the gastric fundus to improve acoustic coupling and visualization of the intramural GV. Blood flow into the GV was confirmed using color doppler. The GVs were followed to try to identify the feeder vessel. Next, either from the distal esophagus (transesophageal transcrural approach) or from the stomach (transgastric approach) the feeder vessel was selectively targeted. If the feeder vessel could not be identified, then the largest intramural part of the GV was targeted. The 19G FNA needle (Echotip, Cook Medical, Winston-Salem, NC) or the Expect Slimline (Boston Scientific, Marlborough, Massachusetts, USA) was used to puncture the GV. After puncturing the GV, the stylet was removed and a 20ml negative-pressure syringe was used to draw blood confirming correct intravascular position. The syringe was then flushed using 5ml saline solution to prevent blood clotting. Embolization coils (MReye or Nester Embolization coils, Cook Medical) were delivered into the varix through the FNA needle using stylet as a pusher. The diameter of the coil was selected based on the diameter of the GV as measured by EUS (typically the size of the coil was chosen to be slightly larger than the size of the GV). Finally, 1-2ml of N-butyl 2 cyanoacrylate (glubran2, GEM Srl, Viareggio, Italy) was injected slowly into the GV after coil deployment followed by 1ml of saline solution to flush the CYA out of the catheter. In the EUS-guided CYA-only patients, only CYA was injected without coil deployment. Doppler flow was used after treatment to confirm absent flow in the treated varix. The echoendoscope was then exchanged for gastroscope to assess for any immediate post procedure complications. **Figure 1** and video-1 show the steps involved in the procedure.

Technical success was defined as the ability to perform the pre-planned therapeutic intervention (EUS-guided CYA injection with or without coiling) successfully. GV obliteration was defined as absent blood flow in the GV confirmed by color doppler flow. This was done immediately post procedure and confirmed during follow-up examination. Repeat endoscopy and EUS were performed at 1-3 months post index procedure followed by regular surveillance endoscopy once GV obliteration is confirmed.

Patients that underwent the procedure for primary or secondary prophylaxis were discharged home after 2 hour observation in the recovery room. Patients that underwent the procedure for active bleeding were kept in hospital to receive appropriate medical therapy for variceal GI bleeding.

## Results

### Demographics

In total, there were 15 patients included in this study. The majority of patients were males (80%) with mean age of 58 ± 12 years. The majority of patients had cirrhosis as the etiology of portal hypertension (86.7%), had Child-Pugh class B and with mean MELD score of 12. The main indication for EUS-guided therapy of the GV was active bleeding or stigmata of recent bleed (53.3%). Baseline characteristics are shown **Table 1**.

**Table 1:** Patient characteristics (n=15).

Characteristic	Value
Mean age, year (+/-SD)	58 (12)
Gender Male, n(%) Female, n(%)	12 (80%) 3 (20%)
Portal hypertension etiology, n(%) Hepatitis B Hepatitis C Alcoholic cirrhosis Autoimmune hepatitis Non-alcoholic steatohepatitis (NASH) Portal vein thrombosis Bilharzia	2 (13.3%) 3 (20.0%) 3 (20.0%) 1 (6.7%) 4 (26.7%) 1 (6.7%) 1 (6.7%)
Hemoglobin level, mean(SD)	10.8 (1.8)
Platelet count, mean (SD)	92 (31.8)
INR, mean (SD)	1.43 (0.22)
MELD score, mean (SD)	12.1 (3.0)
Child Pugh Score, mean (SD)	6.7 (1.1)
Child Pugh Classification, n(%) A B C	7 (46.7%) 8 (53.3%) 0 (0%)
Concomitant hepatocellular carcinoma	2 (13.3%)
Beta Blocker use before endoscopic therapy	8 (53.3%)
Bleeding at time of endoscopic therapy, n(%) Active Primary prophylaxis Secondary prophylaxis	8 (53.3%) 2 (13.3%) 5 (33.3%)

### Procedure

The most common type of GV was GOV-2 (Based on Sarin classification) with mean size of 24.9 ± 7.9mm. Most procedures were done under conscious sedation only (80%). All procedures were technically successful. Most patients underwent combined EUS-guided coiling and CYA-injection and only minority of patients undergoing EUS-guided CYA-injection alone (80% vs. 20%, respectively). The mean number of coils used was 1.5 ± 1.4 coils and the mean volume CYA injected was 1.5 ± 0.74ml. Procedural data are shown in **Table 2**.

**Table 2:** Procedure and follow up data.

Characteristic	Value
Varix type (Sarin classification), n(%) IGV-1 GOV-2	3 (80%) 12 (20%)
Varix size (mm), mean(SD)	24.9 (7.9)
Coils used, mean (SE)	1.5 (1.4)
Glue volume (ml), mean (SD)	1.5 (0.74)
Technical success, n (%)	15 (100%)
Procedure sedation, n(%) Conscious sedation General anesthesia	12 (80%) 3 (20%)
Adverse events, n(%) Pain Embolization Late GV re-bleeding Fever Infection	1 (6.7%) 0 (0%) 0 (0%) 0 (0%) 0 (0%)
Treatment sessions, median (range)	1 (1-2)
Follow-up duration in days ,mean (SD)	174.3 (221.7)
EUS confirmed obliteration of GV, n(mean)	15 (100%)

### Safety and follow-up

All patients tolerated the procedure well with only 1 patient experiencing moderate post procedure epigastric pain that was managed with oral analgesics as an outpatient. One patient had mild bleeding at the puncture site which stopped after coil deployment. There were no major adverse events (late bleeding, systemic embolization or infection) and no procedure-related mortality was observed. There were no unplanned admission to the hospital post procedure.

During follow-up (mean 174.3± 221.7 days) only one patient was found to have incompletely obliterated GV and underwent a second EUS-guided therapy with coiling and CYA-injection. Follow-up of that patient confirmed GV obliteration after the second treatment session.

Detailed description of the patients and the procedure are shown in **Table 3**.

**Table 3:** Detailed description of the patients.

Characteristic	Patient Number															
	1	2	3	4	5	6	7	8	9	10	11	12	13	14	15
**Gender**	Male	Male	Male	Male	Male	Male	Male	Male	Male	Female	Female	Male	Male	Male	Female
**Age (years)**	68	78	62	43	38	61	53	74	41	45	61	69	58	61	58
**PHT etiology**	AIH	HBV	EtOH	EtOH	EtOH	NASH	NASH	NASH	PVT	HBV	NASH	HCV	Bilharzia	HCV	HCV
**Indication**	Active	Active	Secondary prophylaxis	Active	Active	Active	Secondary prophylaxis	Active	Primary prophylaxis	Active	Active	Secondary prophylaxis	Secondary prophylaxis	Secondary prophylaxis	Primary Prophylaxis
**CPS**	7/B	5/A	5/A	7/B	6/A	7/B	6/A	8/B	6/A	7/B	6/A	9/B	7/B	6/A	8/B
**MELD**	13	10	8	12	10	11	11	11	10	17	9	19	15	11	14
**Total sessions**	1	1	1	1	1	1	1	1	1	2	1	1	1	1	1
**Sedation**	CS	CS	CS	CS	GA	CS	CS	CS	CS	GA	CS	CS	GA	CS	CS
**GV type**	GOV-2	GOV-2	IGV-1	GOV-2	GOV-2	GOV-2	GOV-2	GOV-2	GOV-2	IGV-1	GOV-2	GOV-2	GOV-2	GOV-2	IGV-1
**GV size (mm)**	21	25	20	15	15	26	33	30	25	40	28	10	22	30	33
**No. Coils inserted**	0	1	1	0	1	1	1	2	3	5	1	0	1	2	3
**CYA used (ml)**	3	1	1	1	1	1	2	1	2	2	1	1	1	1	3
**Follow up (days)**	90	35	117	42	607	27	410	97	34	75	45	65	710	233	27

AIH, Autoimmune hepatitis; HBV, Hepatitis B infection; HCV, Hepatitis C infection; EtOH, Alcohol; CPS, Child-Pugh classification; MELD, Model for End-Stage Liver Disease; GV, Gastric varix; CYA, cyanoacrylate; GOV, Gastro-esophageal varices; IGV, Isolated Gastric Varices; PVT, Portal Vein Thrombosis; PHT, Portal hypertension.

## Discussion

The management of bleeding GV is very challenging and treatment options are evolving over time. Currently, societal guidelines recommend endoscopic CYA injection or Transjugular Intrahepatic Portosystemic Shunt (TIPS) as the preferred modalities for the treatment of bleeding GV (10). However, endoscopic CYA injection may not possible if the site of the bleeding is covered with blood, in addition to being associated with a number of severe adverse events including systemic embolization and death (11). Furthermore, TIPS requires specialized interventional radiology expertise and many patients have contraindications to undergoing such procedure limiting its utility as a primary therapy for bleeding GV. More recently, EUS-guided therapy has been introduced as a new and novel endoscopic therapy for GV. This therapeutic intervention entails the delivery of endovascular coils and/or CYA into the GV under real-time ultrasound guidance.

A number of retrospective studies (mostly from North America and Europe) have shown that EUS-guided therapy is highly effective and safe option for the treatment of GV (12, 13). Our study represents the first study that evaluates the safety and efficacy and EUS-guided therapy for GV specifically in Arab population. In our study, EUS-guided therapy was found to be very effective and safe in managing patients with clinically significant GV with a treatment and clinical success of 100%, yet associated with low rate of adverse events (<10%) that were mild in severity. These findings are consistent with the results of 2 recently published meta-analyses which an obliteration rate >90% with EUS-guided therapy coupled with excellent safety profile (9, 14).

In our cohort, patients were selected for EUS-guided therapy if they had clinically significant GV (actively bleeding, stigmata of recent bleed or secondary prophylaxis). One patient was selected for primary prophylaxis as he had large GV (25mm) secondary to idiopathic portal vein thrombosis. This patient underwent EUS-guided coil embolization followed by CYA injection resulting in complete obliteration after a single session. He was subsequently treated successfully with full-dose anticoagulation without bleeding. Of note, we were able to achieve high GV obliteration rate after a single session with the use of small volume CYA injection (mean 1.4ml). This was done by selectively targeting the feeder vessel when possible in addition to using combination therapy with coil embolization which lead to effective GV obliteration with minimal CYA injection. This CYA volume is significantly less than what is usually required when it is delivered by endoscopy alone as shown by Lobo et al. (1.40 ml vs. 3.07 ml, p=0.002 for EUS-guided therapy vs. endoscopic-injection alone, respectively) (15). Since most adverse events are related to CYA injection (16), we were able to minimize adverse events by following this strategy.

When feasible, the majority of patients underwent combination coil embolization followed by CYA injection. The rationale behind the combination therapy is the coil would act as a scaffold that would concentrate the CYA in the varix increasing its effectiveness in addition to preventing CYA embolization, hence reducing the risk of adverse events. Bhat et al. has shown that this strategy can achieve GV obliteration rate of 93% (8). Furthermore, a recent meta-analysis concluded that combination EUS-coil-CYA has significantly higher technical and clinical success compared to EUS-CYA alone and EUS-coil alone strategies (17). More importantly, the same meta-analysis has shown that the adverse events were significantly lower with EUS-CYA-coil compared to EUS-CYA alone and has similar safety profile to EUS-coil alone. The only 3 patients in our cohort that underwent EUS-CYA injection alone had on average smaller GV (10mm, 15mm and 21mm) which made deployment of coils technically difficult. Therefore, when technically feasible, we prefer to use the combined-therapy approach to increase our success rate and potentially minimize adverse events.

The safety profile of EUS-guided therapy is quite remarkable in our study in concordance with previously published studies (9). We did not experience any major adverse events such as systemic embolization or infection after a mean follow-up of 174 days. One patient experienced moderate post-procedure epigastric pain that was managed with oral analgesics on outpatient basis. Interestingly, this patient had large GV (40mm) that required 4 coils and 1 ml CYA injection during the first treatment session. This pain may represent ischemia of the large GV in relation to successful obliteration of the GV. Hence larger GV and the need for multiple coils may predict which patients that are likely to experience post-procedure pain, but larger studies are needed to confirm this observation. One patient had mild puncture-site bleeding (no drop in hemoglobin or change in hemodynamics) during the procedure which ceased after coil deployment. Hence our study confirmed the safety profile of EUS-guided GV therapy and strongly favors it as an initial intervention for the management of GV over endoscopic CYA injection alone.

Our study has a number of limitations. It is a retrospective, single-center study with all the adherent potential biases that come with retrospective studies including recall bias and missing data. However, we minimized this risk by keeping a well-maintained database of all patients undergoing EUS-guided interventions. Our study is based on a small sample size (15 patients) limiting its statistical power. However, it represents our initial experience of such intervention and the first experience in Arab population increasing its clinical significance. Finally, the study was conducted in a single-center and all procedures were performed by a single endoscopist limiting the generalizability of our findings.

In summary, we report our initial experience with EUS-guided therapy for GV in Arab population. It appears to be very effective and extremely safe in patients with portal hypertension and clinically significant GV. Further multi-center prospective comparative studies are needed to compare the effectiveness of EUS-guided therapy to endoscopic-CYA injection in patients with GV. Furthermore, cost-effectiveness studies will also be of value to understand the economic value of such intervention in comparison to endoscopic therapy.

## Data availability statement

The raw data supporting the conclusions of this article will be made available by the authors, without undue reservation.

## Ethics statement

The studies involving human participants were reviewed and approved by Ministry of Health, Kuwait. Written informed consent for participation was not required for this study in accordance with the national legislation and the institutional requirements.

## Author contributions

AAA: Study concept and design; acquisition of data; analysis and interpretation of data; drafting and revision of the manuscript; AH: Study concept and design; drafting and revision of the manuscript; AA: Study concept and design; drafting and revision of the manuscript. All authors approved the final version of this manuscript.

## Conflict of interest

The authors declare that the research was conducted in the absence of any commercial or financial relationships that could be construed as a potential conflict of interest.

## Publisher’s note

All claims expressed in this article are solely those of the authors and do not necessarily represent those of their affiliated organizations, or those of the publisher, the editors and the reviewers. Any product that may be evaluated in this article, or claim that may be made by its manufacturer, is not guaranteed or endorsed by the publisher.

## Supplementary material

The Supplementary Material for this article can be found online at: https://www.frontiersin.org/articles/10.3389/fgstr.2022.989987/full#supplementary-material


